# Association between social support and health-related quality of life among Chinese seafarers: A cross-sectional study

**DOI:** 10.1371/journal.pone.0187275

**Published:** 2017-11-27

**Authors:** Jing Xiao, Binjun Huang, Huan Shen, Xiuli Liu, Jie Zhang, Yaqing Zhong, Chuanli Wu, Tianqi Hua, Yuexia Gao

**Affiliations:** 1 Department of Epidemiology and Medical Statistics, School of Public Health, Nantong University, Nantong, Jiangsu, China; 2 Department of Health Promotion, Wuxi Center for Disease Control and Prevention, Wuxi, Jiangsu, China; 3 Department of Health, Nantong Entry-Exit Inspection and Quarantine Bureau, Nantong, Jiangsu, China; 4 Department of Health Management, School of Public Health, Nantong University, Nantong, Jiangsu, China; Xi'an Jiaotong University School of Medicine, CHINA

## Abstract

**Background:**

Seafarers have reported impaired health and health-related quality of life (HRQOL). Social support might increase HRQOL, but little is known about this association among Chinese seafarers. The aim of this study was to describe social support and explore its association with HRQOL among Chinese seafarers.

**Methods:**

A cross-sectional survey was conducted in the ports of Nantong and Rugao, China, from April to December 2013. A total of 917 Chinese seafarers were interviewed on social support, mental distress, perceived occupational stress, and HRQOL using the following self-administered questionnaires: The Social Support Rating Scale, Self-rating Depression Scale, Occupational Stress Questionnaire, and the World Health Organization Quality of Life-BREF (WHOQOL-BREF) questionnaire. Hierarchical linear regression modelling was used to analyze the association between seafarers’ subjective level of social support and their HRQOL.

**Results:**

Of the 917 male Chinese seafarers included in the study, 40.7% perceived high levels of social support, and 39.1% were highly satisfied with their overall quality of life (QOL). Hierarchical regression analysis showed significant associations between level of social support and all health dimensions in the WHOQOL-BREF, even after adjusting for depressive symptoms, occupational stress, occupational activities, sleep duration, and other relevant covariates. Compared with the medium or low level social support group, seafarers with a high level of social support had better QOL scores in the general facet health and QOL (*β* = 2.43, *p*<0.05), and the physical health (*β* = 3.23, *p*<0.001), psychological health (*β* = 5.56, *p*<0.001), social relation (*β* = 6.07, *p*<0.001), and environment domains (*β* = 4.27, *p*<0.001). In addition, depression, occupational stress, occupational activities, and sleep duration were found to be determinants of seafarers’ HRQOL.

**Conclusions:**

Chinese seafarers have poorer HRQOL than the general population, but social support has a significant positive effect on their HRQOL. Efforts to improve social support should be undertaken.

## Introduction

Seafaring is recognized as a risky occupation with higher morbidity and mortality rates than land-based industries [1]. The mortality rate among seafarers on Danish merchant ships was more than six times that of workers employed ashore in 2002–2009 [2]. The rate of suicide committed at work among seafarers was substantially higher than among the general British population from 1919 to the 1970s [3]. Many studies have reported hearing and visual impairment; obesity; and diseases of the circulatory, digestive, and musculoskeletal systems among seafarers [4–6]. Shipping accidents, confined work environments, high consumption of alcohol and tobacco, unhealthy diet, and inadequate physical activity are the major contributing factors to these health problems [7–10]. Health risks are exacerbated by stress, related to the highly demanding psychological and physical working conditions, including long-term separation from family, isolation, long working hours, heat in the workplace, hard physical work, and lack of sleep [11]. These factors may result in seafarers experiencing unique physiological and psychological health problems.

Numerous studies have centered on seafarers’ physical health and its potential determinants, while the psychological well-being of seafarers is an important problem, but few studies have focused on this. Generally, seafarers’ mental health and health-related quality of life (HRQOL) is poor. A burnout syndrome survey conducted among 264 German seafarers indicated that 10.8% of participants experienced emotional exhaustion, 14.0% were at risk of depersonalization, and 62.2% had a low sense of personal accomplishment [12]. Higher work demands significantly affected the general mental health and perceived stress of Swedish seafarers (*n* = 685) [13]. A study of 162 seafarers in an Italian shipping company demonstrated that engineer officers felt more anxiety and greater dissatisfaction with their working life compared with deck crew [14]. A survey of Chinese seafarers, using the SCL-90 scale, found that the mental health of seafarers was below the Chinese norm, and that positive coping style and social support were related to better mental health [15–16].

A new challenge faced by mariners is the rampant return of piracy, which is likely to make affected seafarers experience stress, anxiety, mental distress, which may result in psychological disorder(s). Thus, both seafarers’ mental and physical health should receive more attention.

Quality of life (QOL) is a useful concept that is widely used for comprehensive assessment of health, although not commonly in the seafaring population. QOL is described as the degree to which persons perceive themselves able to function physically, emotionally, mentally, and socially [17]. The World Health Organization (WHO) defined QOL as an “individual’s perception of life in the context of the culture and value system in which they live and in relation to their goals, expectations, standards and concerns” [18]. QOL can be determined by a wide range of factors, including demographic characteristics, health behaviors, (depressed) mood, and social support [19–21]. Social support is defined in terms of social network characteristics—such as assistance from family, friends, neighbors, and other community members—that help individuals to cope with everyday life, particularly in response to critical situations [22–23]. Social support could be an important contributor to improve health and HRQOL among elderly people and individuals with chronic diseases [24–27]. Positive emotional and informational support that may normally be provided is important in maintaining HRQOL [28]. Perceived support seems to be most important [29], especially when stress is experienced [30]. Seafaring workers are usually exposed to mental, psychosocial, and physical stressors that are related to the different duties onboard ships [31]. Little is known about whether social support among seafarers could help them cope with their harsh living conditions and adapt to their environment and lifestyle, as little attention has been focused on the association between social support and QOL of people employed at sea. The aim of this study was to identify current subjective QOL of seafarers and to highlight the relationship between social support and QOL domain scores. Additionally, we assessed the contribution of depressive symptoms, occupational stress, occupational activities, and sleep duration on various dimensions of subjective QOL.

## Methods

### Study population

This cross-sectional study was conducted in the ports of Rugao and Nantong, China, from April to December 2013. The study protocol was approved by the Affiliated Hospital of Nantong University’s Committee on Human Experimentation. All participants provided written informed consent.

A cluster sampling strategy was employed to select participants from the Nantong Entry-Exit Inspection and Quarantine Bureau (NTCIQ). Before the health quarantine inspection, captains of the ships were trained to administer the questionnaire and crew members were invited to complete the questionnaire. Since 98% of investigated seafarers were male and the shipboard working situation probably differs by gender, only male seafarers were included. Of the 929 seafarers who consented to participate in the study, 12 questionnaires were voided because participants did not complete more than 20% of the items of the WHO Quality of Life-BREF (WHOQOL-BREF) questionnaire, as recommended by the WHOQOL Group [32]. Thus, 917 (98.7%) valid questionnaires were evaluated.

### The World Health Organization Quality of Life-BREF

Subjective QOL was the dependent variable, assessed by means of the Chinese version of the WHOQOL-BREF questionnaire [33], a 26-item self-administered generic questionnaire that is a short version of the WHOQOL-100 scale (S1 Questionnaire). Responses range from 1 (very dissatisfied/very poor) to 5 (very satisfied/very good). This instrument emphasizes the subjective response of individuals rather than objective life conditions, with assessment made over the preceding two weeks. It comprises domains and subdomains (facets). The items on ‘‘overall rating of QOL” and “subjective satisfaction with health”, are not included in the domains, but are used to constitute the facet on QOL and general health (general facet). The more popular model for interpreting the scores of WHOQOL-BREF has four domains, namely, physical health (seven items), psychological health (six items), social relations (three items), and environment (eight items). Our analysis was based on this model. The domain scores of the WHOQOL-BREF can be computed according to WHO guidelines [32]. The mean score of items within each domain is used to calculate the domain score. Mean scores are then multiplied by 4 in order to make domain scores comparable with the scores used in the WHOQOL-100, and are subsequently transformed to a scale from 0–100. In order to understand the status of the seafarers’ subjective satisfaction, we employed the definition of subjects’ satisfaction as the level of positive appreciation for each item of the WHOQOL-BREF. Previous studies [34–36] graded the levels of satisfaction for each item according to the percentage of respondents who positively appreciated the item, as follows: dissatisfaction (<50%), bare satisfaction (50–65%), moderate satisfaction (66–74%), and highest satisfaction (≥75%). The WHOQOL-BREF has adequate internal consistency and reliability, and the α-coefficient was 0.92 in this study.

### Social support scale

The Social Support Self-Rating Scale (SSRS) (S1 Rating Scale) developed by Xiao is one of most commonly used instruments for measuring social support in China [37]. It consists of 10 items measuring three dimensions: subjective support (four items), objective support (three items), and support-seeking behavior (three items). The SSRS scale can be used in two ways. First, raw scores within each of the three domains are summed, giving a subjective (range: 8–32) and objective (range: 1–22) support score, and a support-seeking behavior score (range: 3–12). Second, item scores are simply added up, generating a total support score ranging from 12–66. This total support score is classified into three categories: low (≤22), moderate (23–44), and high (≥45) levels of support. Our analysis was based on the second model. The internal consistency of the SSRS scale in this study was acceptable (α-coefficient = 0.76).

### Depression symptom scale

Symptoms of depression were evaluated using the 20-item Zung Self-Rating Depression Scale (SDS) (S2 Rating Scale) [38]. Subjects rate each item with regard to how frequently they experienced each symptom during the preceding week; responses range from 1 (none or seldom) to 4 (most or constant), and 10 items are reverse scored. A raw score is produced and converted into a self-rating score, interpreted as follows: normal range (≤49), mild (50–59), moderate (60–69), and severe (≥70) depression. This self-rating questionnaire is widely used and has satisfactory reliability and validity; the α-coefficient was 0.84 in this study.

### Occupational characteristics

Occupation-related factors included sailing duration, working area, position, class, occupational physical activity (OPA), and work-related psychosocial stress. OPA was calculated as the time spent engaging in physical activity at work per week divided into three groups: light, moderate, and vigorous OPA. OPA levels were classified as energy expenditure per week, expressed in metabolic equivalent tasks (MET)-minutes per week (MET-min·wk^-1^) [39]. MET scores of 1.5, 4.0, and 8.0 MET-hour·wk^-1^ were classified as light, moderate, and vigorous activity, respectively, and an index was calculated by summarizing the MET-hour·wk^-1^ for each physical activity’s intensity level [40].

Work-related psychosocial stress has been shown to decrease seafarers’ psychological well-being [13, 21], it may also impact their HRQOL. A self-administered scale was designed, to measure work-related psychosocial stress, based on research and interviews with experts (S2 Questionnaire) [11, 31, 41]. It comprised 6 items describing psychosocial stress, including concern/anxiety about weather conditions, ship safety, occupational strain, port state control, sea piracy, and family members. Respondents rated each item with regard to how frequently they experienced such concerns during the preceding week using a 3-point Likert scale from 1 (low anxiety) to 3 (severe anxiety). These scores were summed to generate a total score, designated as the “psychosocial stress” variable. The scale has good reliability with an α-coefficient of 0.84. Each item had an α-coefficient ranging from 0.80 to 0.83.

### Socio-demographic characteristics

Demographic characteristics included age, marital status, educational level, residence, body mass index (BMI), self-rated health (SRH), sleep duration, and leisure time physical activity (LPA). LPA was calculated as the time spent engaging in leisure activities (such as walking, table tennis, or tai chi) per week on board.

The Chinese version of the whole questionnaire were showed in “S3 Questionnaire”. The whole dataset were showed in S1 Data.dta.

### Statistical analysis

Analysis was conducted using Stata Statistical Software, Release 12 (College Station, TX: StataCorp LP). Data are presented as mean ± standard deviation (SD) for continuous variables and as the number (percent) for categorical variables. First, we assessed the level of satisfaction with the items, using the operational definitions described earlier. Second, the differences between variables for social support were analyzed using independent sample *t*-tests for continuous variables, χ^2^ tests for categorical variables, and Wilcoxon-Mann-Whitney rank sum test for rank variables. Thirdly, hierarchical regression models were employed to explore the association between different levels of social support and HRQOL.

The ordinary least squares (OLS) regression model was constructed as follows:

$$Y=\beta_{0}+\beta_{1}\;X_{s}+\beta_{2}X_{2}+\beta_{3}X_{3}+\varepsilon$$

where Y is the measure of the general facet on QOL and health, and the physical health, psychological health, social relation, and environment domain for individuals; X_s_ (social support) is the key independent variable, as the subscales of social support were highly correlated with one another, so the different levels of social support divided by the SSRS total score were used to avoid problems of multicollinearity; X_2_ is a vector of other independent variables including depression, occupational characteristics, sleep duration, and LPA; X_3_ is set of dummy covariate variables including marital status, educational level, residence, BMI, and SRH with the exception of age and sailing duration; OPA, LPA, sleep duration, and sailing duration had right-tailed distributions, so the natural log of these four variables were used in the regression model; and ε is an unobserved error term. The coefficient β_1_ is our focused parameter. All statistical tests were two-tailed, and *p*<0.05 was regarded as being statistically significant.

## Results

### Participant characteristics

In 917 male participants (Table 1), the total SSRS mean score was 42.2 (SD 7.0), with 0.5%, 58.8%, and 40.7% of participants categorized as having low, moderate, and high levels of social support, respectively. The mean age of participants was 33.5 years; 55.1% had junior college education or above; 67.8% were from rural areas; the mean sailing duration was 91.6 months; 42.7% worked in the deck department; and 57.3% were ordinary crew, with no differences between the high support group and low or moderate support group. The groups differed in terms of SRH, LPA, psychosocial stress, depression, and HRQOL scores. Participants in the high support group had better SRH and HRQOL, were engaged in more LPA, had lower psychosocial stress, and a lower incidence of depressive symptoms. Regarding depressive symptoms, 51.0% of participants scored in the normal range, with 23.2%, 24.1%, and 1.6% scoring in the mild, moderate, and severe depression range. In the study, respondents felt moderately or severely nervous about: their financial situation (55.4%), ship safety (64.3%), occupational strain (52.7%), port state control (51.5%), sea piracy (59.5%), and family members (66.5%).

**Table 1:** Characteristics of all participants by level of social support.

Variable	Total	Level of social support		*p*-value
		Low/moderate	High	
Age (years)	33.5±9.6	33.3±9.6	33.8±9.5	0.455^*a*^
Education				
Pre-college	412 (44.9)	246 (45.2)	166 (44.5)	0.609^*b*^
Junior college	417 (45.5)	242 (44.5)	175 (46.9)	
Bachelor's degree or above	88 (9.6)	56 (10.3)	32 (8.6)	
Marital status				
Single/separated/divorced/widow	336 (36.6)	219 (40.3)	117 (31.4)	0.006^*b*^
Married	581 (63.4)	325 (59.7)	256 (68.6)	
Residence				
Rural	622 (67.8)	357 (65.6)	265 (71.1)	0.084^*b*^
City	295 (32.2)	187 (34.4)	108 (28.9)	
Working area				
Deck department	392 (42.7)	235 (43.2)	157 (42.1)	0.784^*b*^
Engineering department	374 (40.8)	217 (39.9)	157 (42.1)	
Others	151 (16.5)	92 (16.9)	59 (15.8)	
Positions classes				
Ordinary crew	525 (57.3)	309 (56.8)	216 (57.9)	0.739^*b*^
Senior officer	392 (42.7)	235 (43.2)	157 (42.1)	
Self-rated health				
Good/very good	557 (60.7)	262 (48.2)	295 (79.1)	<0.001^*b*^
Fair	319 (34.8)	246 (45.2)	73 (19.6)	
Bad/very bad	41 (4.5)	36 (6.6)	5 (1.34)	
Prevalence of depression	489 (44.6)	316 (58.1)	133 (35.7)	<0.001^*b*^
Sailing duration (months)	91.6±100.2	89.4±97.6	94.7±104.1	0.600^*c*^
BMI (kg/m^2^)	23.4±2.9	23.4±2.9	23.5±2.8	0.523^*c*^
Sleep duration (hours)	8.2±1.5	8.0±1.5	8.3±1.5	0.003^*c*^
LPA per week (hours)	2.5±4.5	2.1±4.0	3.2±5.1	<0.001^*c*^
OPA per week (MET-hour)	93.8±117.6	94.0±114.6	93.5±122.2	0.211^*c*^
Psychosocial stress (score)	10.2±2.9	10.5±3.0	9.7±2.7	<0.001^*a*^
WHOQOL-BREF (score)				
General facet on QOL and health	56.4±18.7	52.8±19.4	61.8±16.4	<0.001^*a*^
Physical health	67.8±12.7	64.6±12.5	72.5±11.4	<0.001^*a*^
Psychological health	64.3±14.8	59.8±14.2	70.8±13.2	<0.001^*a*^
Social relation	63.8±17.1	59.4±17.5	70.2±14.2	<0.001^*a*^
Environmental domain	52.5±16.6	48.4±16.1	58.4±15.6	<0.001^*a*^
Sample size	917	373	544	

Data are presented as mean ± standard deviation or n (%); BMI, body mass index; LPA, leisure time physical activities; OPA, occupational physical activities; QOL, quality of life

^*a*^ independent sample t-test

^*b*^ Chi-square test

^*c*^ Wilcoxon-Mann-Whitney rank sum test

### Satisfaction with circumstances of life

Using the criteria previously defined, seafarers were generally not satisfied with their life circumstances; only 39.1% participants were satisfied with their overall QOL (Table 2). Less than 50% of participants identified 15 items (of the total 26 items) with which they felt satisfaction. Participants were least satisfied with the availability of money for their needs (16.9%), followed by leisure activity opportunities (18.3%), availability of information (22.9%), and the environment (27.9%). Only 50–65% of participants felt satisfied with their duration of sleep, accommodation, and health. Participants were highly satisfied with their work capacity. Meanwhile, urgent demand for medical treatment (82.1%) and dissatisfaction with the accessibility of the health service (45.4%) were reported.

**Table 2:** Level of group satisfaction with QOL items: WHOQOL-BREF.

Level of satisfaction			
Highest satisfaction (≥75%)	Moderate satisfaction (66–74%)	Bare satisfaction (50–65%)	Dissatisfaction (<50%)
- Need for medical treatment* (82.1%) - Work capacity (75.6%)	- Ability to get around (71.5%) - Support from friends (70.8%) - Activities of daily living (70.2%) - Self-satisfaction (70.2%) - Personal relations (68.7%) - Negative feeling* (68.2%)	Satisfaction with: - health (63.7%) - accommodations (59.6%) - sleep (55.3%)	- Satisfaction with sex (48.9%) - Ability to concentrate (46.7%) - Accessibility of the health service (45.4%) - Physical pain* (44.9%) - Transport (44.5%) - Meaningful life (43.7%) - Feeling safe (43.4%) - Energy (41.8%) - Bodily appearance (40.1%) - Overall QOL (39.1%) - Enjoyment of life (38.1%) - Environment (27.9%) - Information available for daily needs (22.9%) - Opportunity for leisure activity (18.3%) - Money (16.9%)

QOL, quality of life; WHOQOL-BREF; World Health Organization QOL-BREF

Satisfaction was defined as ≥50% of participants regarded the item as good/very good; dissatisfaction was defined as <50% of participants regarded the item as good/very good.

* Reverse scoring

### Social support and health-related QOL

The OLS regression analysis revealed a significant association between social support and HRQOL (Tables 3 and 4). In Model 1, high-level social support was significantly associated with all domains of the WHOQOL-BREF, as well as the general facet QOL and health. Social support explained 8.6–13.1% of the total variance among the four domains and 5.4% of the total variance in the general facet QOL and health. However, social support explained a larger share of the total variance in the psychological health (R^2 =^ 13.1%) than the other domains (physical health, R^2 =^ 9.4%; social relations, R^2 =^ 9.6%; and environment domain, R^2 =^ 8.6%). After including the other independent variables and adjusted for covariates (Model 5), the association between social support and HRQOL persisted (*p*<0.05). Compared with the group with moderate or low levels of social support, seafarers with high levels of social support had better QOL scores in the general facet health and QOL (*β =* 2.43, *p<*0.05), physical health (*β =* 3.23, *p*<0.001), psychological health (*β =* 5.56, *p<*0.001), social relations (*β =* 6.07, *p*<0.001), and environment domain (*β =* 4.27, *p*<0.001). Model 5 showed that 24.0% (social relation) to 32.9% (physical health and psychological health) of the total variance in the different health domains and the general facet on QOL and health was explained by social support.

**Table 3:** Association between social support and the general facet on QOL and health.

	Model 1	Model 2	Model 3	Model 4	Model 5
High social support	8.98***	7.28***	6.23***	5.73***	2.43**
(Low and moderate = 0)	[1.19]	[1.21]	[1.18]	[1.18]	[1.15]
Depressive symptoms		-7.59***	-7.37***	-6.61***	-4.58***
(No = 0)		[1.23]	[1.20]	[1.21]	[1.15]
Psychosocial stress			-1.13***	-1.08***	-0.46**
			[0.21]	[0.21]	[0.21]
ln OPA			-1.58***	-1.54***	-1.13***
			[0.35]	[0.35]	[0.34]
Engineering department			0.88	1.11	0.56
(Deck department = 0)			[1.27]	[1.25]	[1.17]
Others			1.48	1.80	1.39
			[1.72]	[1.71]	[1.73]
Senior officer			-0.01	0.03	1.26
(ordinal crew = 0)			[1.24]	[1.24]	[1.33]
ln sleeping duration				13.68***	9.49***
				[3.37]	[3.22]
ln LPA				0.72	0.42
				[0.66]	[0.61]
Constant	52.78***	57.19***	74.10***	43.96***	36.11***
	[0.83]	[1.05]	[2.76]	[7.72]	[11.50]
Control variables	no	no	no	no	yes
Observations	917	917	917	917	917
Adjusted R^2^	0.054	0.092	0.144	0.158	0.264

QOL, quality of life; ln, natural logarithm; OPA, occupational physical activity; LPA, leisure-time physical activity; Note: robust standard errors in brackets

*** *p*<0.01

** *p*<0.05

* *p*<0.1

Health-related quality of life (HRQOL) was entered as the outcome variable, while social support was entered as the independent variable in Model 1. The depression variable was added to create Model 2, and to explore the association among social support, depression, and HRQOL. Occupational characteristic variables were added to Model 2 to create Model 3; then health-related variables (sleep duration and LPA) were added to create Model 4. Finally, Model 4 was adjusted for control variables, to create Model 5. The control variables were age at interview, education, marital status, self-rated health, and body mass index.

**Table 4:** Associations between social support and four domains of HRQOL.

	Physical health domain		Psychological health domain		Social relations domain		Environment domain	
	Model 1	Model 5	Model 1	Model 5	Model 1	Model 5	Model 1	Model 5
High social support	7.93***	3.23***	11.00***	5.56***	10.8***	6.07***	9.98***	4.27***
(Low and medium = 0)	[0.80]	[0.78]	[0.92]	[0.90]	[1.05]	[1.04]	[1.06]	[1.03]
Depressive symptoms		-4.60***		-6.55***		-4.40***		-2.75***
(No = 0)		[0.76]		[0.89]		[1.05]		[0.97]
Psychosocial stress		-0.95***		-0.71***		-0.86***		-1.47***
		[0.13]		[0.15]		[0.19]		[0.17]
ln OPA		-0.37*		-0.34		-0.76**		-0.83***
		[0.22]		[0.26]		[0.32]		[0.28]
Engineering		0.61		0.0039		0.75		-0.98
(Deck department = 0)		[0.76]		[0.89]		[1.10]		[1.00]
others		0.07		-0.07		3.30**		2.40*
		[1.06]		[1.28]		[1.52]		[1.40]
Senior officer		0.88		0.14		2.90**		2.83**
(ordinal crew = 0)		[0.91]		[1.07]		[1.24]		[1.21]
ln sleeping duration		5.59**		2.14		2.93		8.17***
		[2.20]		[2.70]		[2.83]		[2.75]
ln LPA		0.45		0.58		0.12		1.07**
		[0.39]		[0.46]		[0.55]		[0.50]
Constant	64.6***	70.20***	59.8***	66.80***	59.4***	62.70***	48.4***	34.50***
	[0.54]	[7.92]	[0.61]	[9.78]	[0.75]	[11.0]	[0.69]	[10.4]
Control variables	No	Yes		Yes		Yes		Yes
Observations	917	917	917	917	917	917	917	917
Adjusted R^2^	0.094	0.329	0.131	0.329	0.096	0.240	0.086	0.318

HRQOL, health-related quality of life; QOL, quality of life; ln, natural logarithm; OPA, occupational physical activity; LPA, leisure-time physical activity; Note: robust standard errors in brackets

*** *p*<0.01

** *p*<0.05

* *p*<0.1

The independent variables in Model 1 and Model 5 were the same as that in Table 3.

The control variables were age at interview, education, marital status, self-rated health, and body mass index.

### Depression and health-related QOL

Depressive symptoms were significantly associated with impaired HRQOL (*p*<0.01). Compared with participants with no depressive symptoms, those with depressive symptoms had decreased QOL scores in the general facet health and QOL (*β =* -4.58, *p<*0.01), and in the physical health (*β =* -4.60, *p<*0.001), psychological health (*β =* -6.55, *p<*0.01), social relations (*β =* -4.40, *p<*0.01), and environment (*β =* -2.75, *p<*0.01) domains. Depressive symptoms explained 3.8% of the total variance in the general facet QOL and health (detailed data: Table 3), and 7.5%, 6.1%, 3.0% and 2.2% in the psychological health, physical health, social relations, and environment domain, respectively.

### Psychosocial stress and HRQOL

Psychosocial stress was a significant independent determinant of HRQOL (*p*<0.05), even after adjusting for other control variables (Tables 3 and 4). For each 1-unit increase in psychosocial stress score, the score of general facet of QOL and health decreased by 0.46 units (*p<*0.05). Similar results were found in four domains: decreased by 0.71 units for the score of psychological domain to 1.47 units for the score of environment domain with each 1-unit increase in psychosocial stress score (both *p<*0.05). Therefore, psychosocial stress exerted a negative effect on HRQOL.

The natural log of OPA had a significant independent negative effect on the general facet QOL and health (*β =* -1.13, *p<*0.001), and the social relations (*β =* -0.76, *p<*0.05) and environment (*β =* -0.83, *p<*0.05) domain. The natural log of sleeping duration was a positive factor for seafarers’ general facet QOL and health (*β =* 9.49, *p<*0.01), physical health (*β =* 5.59, *p<*0.05) and environment domain (*β =* 8.17, *p<*0.01). The natural log of LPA could improve seafarers’ health in the environment domain (*β =* 1.07, *p<*0.05). In addition, seafarers who reported good or very good health also showed higher scores of HRQOL in all domains (*p*<0.01), as well as in the general facet QOL and health (*p*<0.001). Seafarers who were married were more likely than those who were single, separated, divorced, or bereaved to report better QOL in all domains (*p*<0.05) except the social relation domain (data not shown).

## Discussion

To the best of our knowledge, this is the first study to explore the association between social support and HRQOL among seafarers. The main findings show that social support is significantly associated with HRQOL. We also demonstrated that as a group, seafarers were not satisfied with their life circumstances, especially in terms of availability of money for their needs, leisure-time physical activity, medical treatment, and availability of information to meet daily needs. Seafarers were shown to have worse QOL than the general population in all four domains of the WHOQOL-BREF. Their mean scores in the physical health, psychological health, social relations, and environment domains were 67.8, 64.3, 63.8, and 52.5, respectively. In a study among 37,740 Chinese male adults, the respondents reported mean scores in the four domains of 75.0, 67.5, 66.4, and 57.1, respectively [42]. In addition, Chinese seafarers reported poorer QOL in the four domains than Polish seafarers [43].

Among items in the four domains, satisfaction with items in the environment domain was poorer than that in other domains (7/8 items were unsatisfying). Only 16.9% of seafarers were satisfied that the money they earned was enough to meet their needs; their wages were considered inconsistent with their occupation. As the wage gap between seafaring and onshore work decreases, some seafarers (mainly those who registered low level of support) have reported low self-esteem and experience of mental health imbalance [43], which can contribute to distress. Additionally, <25% of participants were satisfied with these two items: leisure-time activities and information acquisition. This may result in increased loneliness and may affect mental health.

In the present study, 49% of the respondents had self-rated depressive symptoms; this rate was much higher than that among the general Chinese population (21.8%) [44], but it was consistent with that in a previous study of seafarers [45]. A large number of qualitative studies have reported that seafarers have poorer mental health with possible associated health damage [46, 47]; the present study provided quantitative evidence for this. We found depressive symptoms had a significantly negative effect on seafarers’ HRQOL (*p*<0.01) after controlling for confounders. In fact, many reports have demonstrated that depressive symptoms are negatively related to a subject’s health and HRQOL in patients and the children [48–50]. Anxiety and depressive mood disorders may impact on seafarers’ mental health; hence, effective prevention programs should be strengthened to improve their psychological wellbeing.

Previous studies showed that seafarers are exposed unique psychosocial stressors, such as diverse climate and long term separation from family, which are related to their ill-health [10, 51]. In this study, we found that seafarers were most worried about family members (66.5%), ship safety (64.2%), and sea piracy (59.5%). A comprehensive literature search indicated that, from 2002–2012, 3,806 ships were attacked and 7,635 incidents involving human victims occurred; this threatens maritime security and health [52]. Ziello et al. assessed psychological consequences of kidnapping in a group of Italian seafarers held in captivity: all the victims showed high scores of anxiety and social adjustment disorder, and, even more seriously, somatic disorders, depression, and post-traumatic stress disorder [53, 54]. Our study robustly confirmed that psychosocial stress at work has an adverse effect on seafarers’ HRQOL. Marriage satisfaction and anxiety levels among seafarers also mediate their work-related stresses and sense of purpose in life [55]. Therefore, more attention should be paid to preventive occupational measures in coping with strain on order to improve the QOL of seafarers.

Social support is an important factor affecting QOL, as has been reported in previous studies. Strine et al. found that inadequate social support was associated with adverse health behaviors, impaired HRQOL, and dissatisfaction with life [56]. Our research confirmed that increased social support was associated with a higher QOL in all domains. According to a social support theory raised by Cobb [57]; Cohen and Willis [58], social support can protect people in crisis from a wide variety of pathological states. The specific character of maritime work has many factors conducive to stress, overload, fatigue, and emotional tension, all of which can negatively influence subjective QOL. Social support could provide a positive effect to improve their health. This study has verified the direct effect of social support among seafarers. However, social support is also a mediator to alleviate the damage caused by stress and depression. Previous research has empirically demonstrated that social support serves as a buffer against the adverse effects of stressors and increases QOL in patients [59, 60]. Maritime work-related stress and depressive symptoms are very common, so the buffering effect of social support should be the subject of further studies.

The strengths of this study include a large sample size in seafaring workers, and a comprehensive data collected to detect association of QOL with social support and its four domains, and extensive information on confounders. However, the major limitation is its cross-sectional design. Therefore, the causal interpretation of associations should be made with caution. In addition, owing to the subjective assessment made by Chinese seafarers in this research, the usual limitations inherent to self-report research are explicit, such as memory bias, reporting bias, common method variance, and social desirability bias. Furthermore, comparatively fewer objective indicators were applied in the presented study and the analysis was based strictly on the subjective indicators of QOL, both the aim of our research and one of the reasons for criticism thereof.

## Conclusions

The study contributes new knowledge about social support and QOL among seafarers. One finding was that Chinese seafarers experienced low QOL and that social support can effectively improve their QOL. Attention should be paid to the status quo of Chinese seafarers. First, base facility construction should be undertaken by shipping companies. Such facilities should include a workers’ exercise center, electronic network equipment, and better medical services, as these would improve the occupational environment of seafarers. Second, emphasis should be placed on the mental health of the crew, providing professional counseling to resolve seafarers’ psychological difficulties. In addition, cultural entertainment activities, interpersonal contact training, or psychological quality training could significantly improve seafarers’ subjective levels of support. The crew themselves, should adjust their mentality, find joy and fulfillment in their careers, and broaden their social interaction, in order to improve subjective levels of support and support utilization. Last, the study findings are an indication to health planners that there is need to focus on proper measures aimed at improving the QOL and occupational conditions of seafarers.

## Supporting information

S1 Questionnaire World Health Organization Quality of Life-BREF (WHOQOL-BREF) questionnaire. (DOCX)

S2 Questionnaire Occupational stress questionnaire. (DOCX)

S3 Questionnaire The Chinese version of the whole questionnaire. (DOCX)

S1 Rating Scale Social Support Rating Scale. (DOCX)

S2 Rating Scale Self-rating Depression Scale. (DOCX)

S1 Data.dta (DTA)
