# Supplementary material for: Association between social support and health-related quality of life among Chinese seafarers: A cross-sectional study
Source: PLoS One. 2017 Nov 27;12(11):e0187275. doi: 10.1371/journal.pone.0187275 (PMC5703501; doi:10.1371/journal.pone.0187275)
Supplement: S1 Questionnaire — (DOCX) [file pone.0187275.s001.docx]

**S1 Questionnaire. World Health Organization Quality of Life-BREF (WHOQOL-BREF) questionnaire**

References: 1. World health organization. WHOQOL user manual. Geneva: WHO 1998.

2. World health organization. WHOQOL user manual. Geneva: WHO 2012.

3. Hao YT, Fang JQ. The introduce and usage of WHOQOL instrument in Chinese. Modern Rehabilitation. 2000; 4: 1127-9, 1145 (in Chinese).
