# Supplementary material for: Association between social support and health-related quality of life among Chinese seafarers: A cross-sectional study
Source: PLoS One. 2017 Nov 27;12(11):e0187275. doi: 10.1371/journal.pone.0187275 (PMC5703501; doi:10.1371/journal.pone.0187275)
Supplement: S2 Questionnaire — (DOCX) [file pone.0187275.s002.docx]

**S2 Questionnaire. Occupational Stress Questionnaire**

In the past month, did you worry about (get nervous for) the following matter in your ordinary jobs?

| whether worry about (get nervous for) the following matter or not | 1. not worried /nervous at all | 2. worried /nervous | 3. extremely worried/nervous |
| --- | --- | --- | --- |
| 1. bad weather conditions (for example, heavy sea and [navigation in fog](https://www.baidu.com/link?url=Q6Hv6E8JHAECnA_PalqsJWUBTPGj8yBYyjS4tP3r6nb0CxSQKjhYsA9pqTV6PRkxIwr-zpB1klBJH2-1awWrCN6_miZOppP9RYcwj1pEAYLxUv4jPdootUr2bYqIYM94&wd=&eqid=8224d4fa000332640000000459352153)) |  |  |  |
| 2. ship safety (for example, mechanical malfunction and etc.) |  |  |  |
| 3. working pressure (for example, leadership criticism and lead inspection) |  |  |  |
| 4. port state control (whether the ship is airworthy, oil pollution inspection) |  |  |  |
| 5. pirate or hijacking event |  |  |  |
| 6. health of family members/children education/ family care and so on |  |  |  |
