# Supplementary material for: Association between social support and health-related quality of life among Chinese seafarers: A cross-sectional study
Source: PLoS One. 2017 Nov 27;12(11):e0187275. doi: 10.1371/journal.pone.0187275 (PMC5703501; doi:10.1371/journal.pone.0187275)
Supplement: S3 Questionnaire — (DOCX) [file pone.0187275.s003.docx]

**S3 Questionnaire. The Chinese version of the whole questionnaire as follows.**

**船员健康状况调查问卷**

**各位船员好：**

远洋船员作为远程海洋作业人群中的一个重要群体，长期生活在远离陆地、饮食单一、工作强度大、工作负担重等特殊的工作环境中，其健康状况及生命质量引起了国内外的医学专家的关注。本研究拟从生理、心理等角度了解您的身心健康状况，为提高海员的身心健康提供政策建议。谢谢您的配合！

问卷编号：

调查时间：

**一、一般情况：**

Al. 年龄： _ (周岁)

A2 性别： 1. 男 2. 女

A3. 文化程度：1. 高中/中专及以下 2. 大专 3. 本科及以上

A4. 婚姻状况： 1. 未婚 2. 已婚 3. 离婚 4. 丧偶 5. 其他

A5. 海龄： A51 年 A52 月

A6. 职业类别： 1. 驾驶 2. 轮机 3. 其他

A7. 驾驶技术职务类别： 1. 水手 2. 三副 3. 二副 4. 大副 5. 船长

A8. 轮机技术职务类别： 1.技工 2.三管轮 3.二管轮 4.大管轮 5.轮机长

A9. A9-1家庭年均收入 万元，A9-2 家庭人口数 人。

A10. 家庭所在地：1. 农村 2.城市

A11. 身高 CM

A12. 体重 公斤。

**二、健康相关行为情况：**

B1 在过去四周，您是否生过病或者受过伤？ 0. 无 1. 有

B2 在过去的一年内，您是否有医生诊断的慢性病？ 0. 否 1. 是

B3 如果有，是那种慢性病？

1. 高血压 2．高血脂 3. 糖尿病或者血糖升高

4. 心脏病 （冠心病、心绞痛、心力衰竭等） 5. 胃炎/胃溃疡

6. 其他

B4. 现在您还在吸烟吗？ 1. 不吸(跳转B5) 2. 吸烟

B4a 如果吸烟，平均每天吸 支

B4b. 吸烟年数： 年

B5. 去年，您曾经喝过啤酒、白酒或别的酒吗？ 1. 没有 2. 喝过

B6. 喝酒的频率：

1. 几乎每天喝 2. 每周3-4次 3. 每周1-2次

4. 每月1-3次 5. 每月少于1次

B7. 饮酒类型和**平均每周**饮酒量

| 酒的种类 | 是否喝酒？  0. 不喝酒 1. 喝酒 | **平均每周**喝多少？ |
| --- | --- | --- |
| 啤酒 | - B7.1a | **□**（瓶）B7.1 |
| 葡萄酒（包括各种果酒、黄酒等） | **□**B7.2a | **□**（两）B7.2 |
| 白酒 | **□**B7.3a | **□**（两）B7.3 |

B8. 包括晚上和白天，你**每天**的睡眠时间有 小时？

B9. 通常每周的工作中有多少时间做这些体力活动？（小时）

1.轻体力劳动（如静坐工作、偶尔站立和坐的工作、办公室工作）： 时

2.中度体力劳动（如技工、电工、长期站立）： 时

3.重度体力劳动（如水手、搬运工、清洁工）： 时

B10. 工作之外时间，业余时间的安排：（就每种活动询问，并填入下表）

| 活动类型 | 是否参加？  0 不参加 1 参加 | 平均**每周**花多少小时？ |
| --- | --- | --- |
| 打乒乓球 | - B10.1a | **□**（小时）B10.1 |
| 散步 | - B10.2a | **□**（小时）B10.2 |
| 看录像/电影，DVD，VCD | **□**B10.3a | **□**（小时）B10.3 |
| 玩电脑游戏/手机游戏/游戏机等 | **□**B10.4a | **□**（小时）B10.4 |
| 读书（报纸、杂志、小说等）、写字或者画画 | **□**B10.5a | **□**（小时）B10.5 |
| 和同事聊天 | **□**B10.6a | **□**（小时）B10.6 |

B11. 在过去的一月内，您平时工作过程中的是否为下列事情担心（紧张）？

| 是否为下列事情担心/紧张？ | 1.不担心/不紧张 | 2.担心/紧张 | 3.非常担心/紧张 |
| --- | --- | --- | --- |
| B11.1天气情况不好（如风浪大、雾航） |  |  |  |
| B11.2 船舶安全（如机器发生故障等） |  |  |  |
| B11.3 工作压力（如领导批评、领导检查） |  |  |  |
| B11.4 港口国检查（船舶是否适航、油污染检查） |  |  |  |
| B11.5 海盗或劫持事件 |  |  |  |
| B11.6 家庭成员的健康/子女教育/亲人照顾等 |  |  |  |

B12. 过去一个月，您总体上的精神状态：

1. 非常愉快 2. 比较愉快 3. 一般 4. 比较压抑 5. 非常压抑

B13. 在过去一年，您觉得您的健康状况如何？

1. 非常好 2. 好 3. 一般 4. 较差 5. 非常差

**三：精神生活情况**

说明：为了全面了解您的健康状况，提高生活质量，特设计了该表。下面有20条文字，请有细阅读每一条，把意思弄明白。1偶或无(**过去一周内**，出现这类情况的日子不超过一天)，2有时(**过去一周内**，有1-2天有过这类情况) ，3经常(过去一周内，3-4天有过这类情况)，4持续(**过去一周内**，有5-7天有过这类情况) 。

|  | 偶尔或无 | 有时 | 经常 | 持续 |
| --- | --- | --- | --- | --- |
| C1. 我觉得闷闷不乐，情绪低沉 |  |  |  |  |
| C2. 我觉得一天之中早晨最好 |  |  |  |  |
| C3. 我一阵阵哭出来或觉得想哭 |  |  |  |  |
| C4. 我晚上睡眠不好 |  |  |  |  |
| C5. 我吃得和平常一样多 |  |  |  |  |
| C6. 我与异性密切接触时和以往一样感到愉快 |  |  |  |  |
| C7. 我发觉我的体重在下降 |  |  |  |  |
| C8. 我有便秘的苦恼 |  |  |  |  |
| C9. 我心跳比平时快 |  |  |  |  |
| C10. 我无缘无故地感到疲乏 |  |  |  |  |
| C11. 我的头脑跟平常一样清楚 |  |  |  |  |
| C12. 我觉得经常做的事情并没有困难 |  |  |  |  |
| C13. 我觉得不安而平静不下来 |  |  |  |  |
| C14. 我对将来抱有希望 |  |  |  |  |
| C15. 我比平常容易生气激动 |  |  |  |  |
| C16. 我觉得做出决定是容易的 |  |  |  |  |
| C17. 我觉得自己是个有用的人，有人需要我 |  |  |  |  |
| C18. 我的生活过的很有意思 |  |  |  |  |
| C19. 我认为如果我死了别人会生活得好些 |  |  |  |  |
| C20. 我感兴趣的事情我仍然照样感兴趣 |  |  |  |  |

**四、社会支持量表**

D1. 您有多少关系密切，可以得到支持和帮助的朋友?(只选一项)

①一个也没有 ②1-2个 ③3- 5个 ④6个及以上

D2. 近一年来您：(只选一项)

1. 远离家人且独居一室 ②住处经常变动，多数时间和陌生人住在一起

③和同学、同事或朋友住在一起 ④和家人住在一起

D3. 您与邻居：(只选一项)

①相互之间从不关心，只是点头之交 ②遇到困难可能稍微关心

③有些邻居都很关心您 ④大多数邻居都很关心您

D4. 您与同事：(只选一项)

①相互之间从不关心，只是点头之交 ②遇到困难可能稍微关心

③有些同事都很关心您 ④大多数同事都很关心您

D5. 从家庭成员得到的支持和照顾（在合适的框内划“√”）

|  | 无 | 极少 | 一般 | 全力支持 |
| --- | --- | --- | --- | --- |
| 1. 夫妻(恋人) |  |  |  |  |
| 1. 父母 |  |  |  |  |
| 1. 儿女 |  |  |  |  |
| 1. 兄弟姊妹 |  |  |  |  |
| 1. 其他成员(如嫂子) |  |  |  |  |

D6. 过去在您遇到急难情况时，曾经得到的经济支持和解决实际问题的帮助的来源有：

①无任何来源。②以下来源：（可选多项）

A. 配偶 B. 其他家人 C. 亲戚 D. 同事 E. 工作单位

F. 党团工会等官方或半官方组织 G. 宗教、社会团体等非官方组织

H. 其它

D7. 过去，在您遇到急难情况时，曾经得到的安慰和关心的来源有：

①无任何来源。 ②以下来源:(可选多项)

A. 配偶 B. 其他家人 C. 亲戚 D. 同事 E. 工作单位

F. 党团工会等官方或半官方组织 G. 宗教、社会团体等非官方组织

H. 其它

D8. 遇到烦恼时的倾诉方式： (只选一项)

①从不向任何人倾诉 ②只向关系极为密切的1-2个人倾诉

③如果朋友主动询问您会说出来 ④主动倾诉，以获得支持和理解

D9. 您遇到烦恼时的求助方式：(只选一项)

①只靠自己，不接受别人帮助 ②很少请求别人帮助

③有时请求别人帮助 ④有困难时经常向家人、亲友、组织求援

D10. 对于团体(如党团组织、宗教组织、工会、学生会等)组织活动，您：(只选一项)

①从不参加 ②偶尔参加

③经常参加 ④主动参加并积极活动

1. **世界卫生组织生活质量调查表(QOL--BREF)**

E1. (GI)总体上说，您怎样评价您的生存质量?

| 很差 | 不好也不差 | 很好 | 好 | 差 |
| --- | --- | --- | --- | --- |
| 1 | 2 | 3 | 4 | 5 |

E2. (G4)您对自己的健康状况满意吗?

| 很不满意 | 不满意 | 既非满意也非不满意 | 满意 | 很满意 |
| --- | --- | --- | --- | --- |
| 1 | 2 | 3 | 4 | 5 |

下面的问题是关于**两周来**您经历某些事情的感觉。

E3. (Fl.4)您觉得疼痛妨碍您去做自己需要做的事情吗?

| 根本不妨碍 | 很少妨碍 | 有妨碍(一般) | 比较妨碍 | 极妨碍 |
| --- | --- | --- | --- | --- |
| 1 | 2 | 3 | 4 | 5 |

E4. (F1.3)您需要依靠医疗的帮助进行日常生活吗?

| 根本不需要 | 很少需要 | 需要(一般) | 比较需要 | 极需要 |
| --- | --- | --- | --- | --- |
| 1 | 2 | 3 | 4 | 5 |

E5. (F4.1)您觉得生活有乐趣吗?

| 根本没乐趣 | 很少有乐趣 | 有乐趣(一般) | 比较有乐趣 | 极有乐趣 |
| --- | --- | --- | --- | --- |
| 1 | 2 | 3 | 4 | 5 |

E6. (F4.2)您觉得自己的生活有意义吗?

| 根本没意义 | 很少有意义 | 有意义(一般) | 比较有意义 | 极有意义 |
| --- | --- | --- | --- | --- |
| 1 | 2 | 3 | 4 | 5 |

E7. (F5.3)您能集中注意力吗?

| 根本不能 | 很少能 | 能 (一般) | 比较能 | 极能 |
| --- | --- | --- | --- | --- |
| 1 | 2 | 3 | 4 | 5 |

E8. (F6.l)日常生活中您感觉安全吗?

| 根本不安全 | 很少安全 | 安全 (一般) | 比较安全 | 极安全 |
| --- | --- | --- | --- | --- |
| 1 | 2 | 3 | 4 | 5 |

E9. (F22.l)您的生活环境对健康好吗?

| 根本不好 | 很少好 | 好 (一般) | 比较好 | 极好 |
| --- | --- | --- | --- | --- |
| 1 | 2 | 3 | 4 | 5 |

下面的问题是关于**两周来您做某些事情的能力**

E10. (F2.1l)您有充沛的精力去应付日常生活吗?

| 根本没精力 | 很少有精力 | 有精力(一般) | 比较有精力 | 完全有精力 |
| --- | --- | --- | --- | --- |
| 1 | 2 | 3 | 4 | 5 |

E11. (F7.1)您认为自己的外形过得去吗?

| 根本过不去 | 很少过得去 | 过得去(一般) | 多数过得去 | 完全过得去 |
| --- | --- | --- | --- | --- |
| 1 | 2 | 3 | 4 | 5 |

E12. (F18.I)您的钱够用吗?

| 根本不够用 | 很少够用 | 够用(一般) | 多数够用 | 完全够用 |
| --- | --- | --- | --- | --- |
| 1 | 2 | 3 | 4 | 5 |

E13. (F20.l)在日常生活中您需要的信息都齐备吗?

| 根本不齐备 | 很少齐备 | 齐备(一般) | 多数齐备 | 完全齐备 |
| --- | --- | --- | --- | --- |
| 1 | 2 | 3 | 4 | 5 |

E14. (F2I.l)您有机会进行休闲活动吗?

| 根本没机会 | 很少有机会 | 有机会力(一般) | 多数有机会 | 完全有机会 |
| --- | --- | --- | --- | --- |
| 1 | 2 | 3 | 4 | 5 |

E15. (F9.1)您行动的能力如何?

| 很差 | 差 | 不好也不差 | 好 | 很好 |
| --- | --- | --- | --- | --- |
| 1 | 2 | 3 | 4 | 5 |

下面的问题是关于**两周来您对自己日常生活各个方面的满意程度**

E16. (F3.3)您对自己的睡眠情况满意吗?

| 很不满意 | 不满意 | 既非满意也非不满意 | 满意 | 很满意 |
| --- | --- | --- | --- | --- |
| 1 | 2 | 3 | 4 | 5 |

E17. (F10.3)您对自己做日常生活事情的能力满意吗?

| 很不满意 | 不满意 | 既非满意也非不满意 | 满意 | 很满意 |
| --- | --- | --- | --- | --- |
| 1 | 2 | 3 | 4 | 5 |

E18. (F12.4)您对自己的工作能力满意吗？

| 很不满意 | 不满意 | 既非满意也非不满意 | 满意 | 很满意 |
| --- | --- | --- | --- | --- |
| 1 | 2 | 3 | 4 | 5 |

E19. (F63)您对自己满意吗?

| 很不满意 | 不满意 | 既非满意也非不满意 | 满意 | 很满意 |
| --- | --- | --- | --- | --- |
| 1 | 2 | 3 | 4 | 5 |

E20. (Fl3.3)您对自己的人际关系满意吗?

| 很不满意 | 不满意 | 既非满意也非不满意 | 满意 | 很满意 |
| --- | --- | --- | --- | --- |
| 1 | 2 | 3 | 4 | 5 |

E21. (F14.4)您对自己的性生活满意吗?

| 很不满意 | 不满意 | 既非满意也非不满意 | 满意 | 很满意 |
| --- | --- | --- | --- | --- |
| 1 | 2 | 3 | 4 | 5 |

E22. (F14.4)您对自己从朋友那里得到的支持满意吗?

| 很不满意 | 不满意 | 既非满意也非不满意 | 满意 | 很满意 |
| --- | --- | --- | --- | --- |
| 1 | 2 | 3 | 4 | 5 |

E23. (F17.3)您对自己居住地的条件满意吗?

| 很不满意 | 不满意 | 既非满意也非不满意 | 满意 | 很满意 |
| --- | --- | --- | --- | --- |
| 1 | 2 | 3 | 4 | 5 |

E24. (F19.3)您对得到卫生保健服务的方便程度满意吗?

| 很不满意 | 不满意 | 既非满意也非不满意 | 满意 | 很满意 |
| --- | --- | --- | --- | --- |
| 1 | 2 | 3 | 4 | 5 |

E25. (F23.3)您对自己的交通情况满意吗?

| 很不满意 | 不满意 | 既非满意也非不满意 | 满意 | 很满意 |
| --- | --- | --- | --- | --- |
| 1 | 2 | 3 | 4 | 5 |

下面的问题是关于**两周来您经历某些事情的频繁程度**。

E26. (F8.l)您有消极感受吗?(如情绪低落、绝望、焦虑、忧郁)

| 没有消极感受 | 偶尔有消极感受 | 时有时无 | 经常有消极感受 | 总是有消极感受 |
| --- | --- | --- | --- | --- |
| 1 | 2 | 3 | 4 | 5 |

您已经认真填完了上表，现在请您对您的总健康状况和生命质量进行总的估计，并按百分制打分(比如78分)，填在下面的横线上。

**E27．您认为您的健康状况和生命质量总评分为： 分(满分为100分)**

**再次谢谢您的合作与支持!**
