# Supplementary material for: Association between social support and health-related quality of life among Chinese seafarers: A cross-sectional study
Source: PLoS One. 2017 Nov 27;12(11):e0187275. doi: 10.1371/journal.pone.0187275 (PMC5703501; doi:10.1371/journal.pone.0187275)
Supplement: S1 Rating Scale — (DOCX) [file pone.0187275.s004.docx]

**S1 Rating Scale. Social Support Rating Scale**

Reference: Xiao, S. The theoretical basis and applications of Social Support Rating Scale (SSRS). J Clinical Psychiatry 1994; (02): 98-100 (in Chinese).

This social support rating scale was widely used in Chinese research, the cited frequency of which was 1861 times in China.

**The translation of this rating scale is as follows.**

**Social Support Rating Scale**

Name: Sex: Age:

Educational Attainments: Occupation: Marital Status:

Address or Organization: Date:

**Instructions:** The following questions are designed to measure your support received in society. Depending on the fact, please finish the rating scale in accordance with the specific requirements of each issue. Thank you for your cooperation.

1. How many intimate friends do you have, from whom you can receive support and help? (Exclusive Choice)
2. None
3. 1~2
4. 3~5
5. no less than 6
6. Over the past year, you (Exclusive Choice)
7. stay away from family, and live alone
8. often move the residence, and most of time live together with strangers
9. live together with students, colleagues or friends
10. live together with family
11. With your neighbors, you (Exclusive Choice)
12. have [a speaking acquaintance](https://www.baidu.com/link?url=Gw7uwil63dC-ikbCRiynD_OLkGQ6zQy4UXn69mtUuaTo7Mpfj9VXyjao3AiOvKgRIDV4I1Q7LEEAbSJhCjcCwWPuk7zl3AisynHEjwYY0Sb4HDMT7yvcYpUUjc0RrNWFzt_vyTgMOkynAzahDOX9-q&wd=&eqid=cbff60e30005a3d7000000045934409b) and never care about each other
13. maybe have a little concern when meeting trouble
14. are deeply concerned by some of them
15. are deeply concerned by most of them
16. With your colleagues, you (Exclusive Choice)
17. have [a speaking acquaintance](https://www.baidu.com/link?url=Gw7uwil63dC-ikbCRiynD_OLkGQ6zQy4UXn69mtUuaTo7Mpfj9VXyjao3AiOvKgRIDV4I1Q7LEEAbSJhCjcCwWPuk7zl3AisynHEjwYY0Sb4HDMT7yvcYpUUjc0RrNWFzt_vyTgMOkynAzahDOX9-q&wd=&eqid=cbff60e30005a3d7000000045934409b) and never care about each other
18. maybe have a little concern when meeting trouble
19. are deeply concerned by some of them
20. are deeply concerned by most of them
21. Obtain support and help from family members (Draw “√” in the suitable box)

|  | none | rarely | normally | full support |
| --- | --- | --- | --- | --- |
| 1. couple |  |  |  |  |
| 1. parents |  |  |  |  |
| 1. children |  |  |  |  |
| 1. siblings |  |  |  |  |
| 1. others (for example, sister-in-law) |  |  |  |  |

1. In the past, when you encounter difficulties, what is the source that you ever received either economic support or practical problem-solving help?
2. no source
3. the following source (more than one answer is permitted)
4. spouse
5. other family members
6. friends
7. relatives
8. colleagues
9. companies
10. official or semi-official organizations, such as, parties, leagues and trade union
11. unofficial organizations, such as, religion, social group and etc.
12. others (please list)
13. In the past, when you encounter difficulties, what is the source that you ever received comfort and caring?
14. no source
15. the following source (more than one answer is permitted)
16. spouse
17. other family members
18. friends
19. relatives
20. colleagues
21. companies
22. official or semi-official organizations, such as, parties, leagues and trade union
23. unofficial organizations, such as, religion, social group and etc.
24. others (please list)
25. What is the way of talking when you are in trouble? (Exclusive Choice)
26. never complain to anyone
27. only complain to 1 or 2 persons who have a close relationship with
28. will talk to the friend who takes the initiative to inquiry
29. take the initiative to talk their own troubles in order to get support and understanding
30. What is the way of seeking help when you are in trouble? (Exclusive Choice)
31. just rely on myself, and do not accept the help of others
32. rarely ask someone for help
33. sometimes ask someone for help
34. ask family, friends or organizations for help when facing troubles
35. Organized activities for groups (such as, party and youth league organizations, [religious organization](https://www.baidu.com/link?url=iv1KeDqu0GjTNfza1L2nbF7Chx4zevhxOnOLUigrlvNds0DmQsHcYdNyr-DUFg76w6JpCKvY2KsRn8kE3_fRGloLe5_vWG024e3Gy5Mkf_PnIyDM40mLFdvtQB6B2RZl&wd=&eqid=f5d2710b0006a8ff000000045934588d), trade union, student union and etc.), you . (Exclusive Choice)
36. never attend
37. occasionally attend
38. often attend
39. take the initiative to attend and are active with
