# Supplementary material for: Association between social support and health-related quality of life among Chinese seafarers: A cross-sectional study
Source: PLoS One. 2017 Nov 27;12(11):e0187275. doi: 10.1371/journal.pone.0187275 (PMC5703501; doi:10.1371/journal.pone.0187275)
Supplement: S2 Rating Scale — (DOCX) [file pone.0187275.s005.docx]

**S2 Rating Scale. Self-rating Depression Scale**

Reference: Zung W. A self-rating depression scale. Arch Gen Psych 1965; 12: 63–70.

The Self-Rating Depression Scale (SDS)

|  | A Little of the Time | Some of the Time | Good Part of the Time | Most of the Time |
| --- | --- | --- | --- | --- |
| 1. I feel down-hearted and blue |  |  |  |  |
| 1. Morning is when I feel the best |  |  |  |  |
| 1. I have crying spells or feel like it |  |  |  |  |
| 1. I have trouble sleeping at night |  |  |  |  |
| 1. I eat as much as I used to |  |  |  |  |
| 1. I still enjoy sex |  |  |  |  |
| 1. I notice that I am losing weight |  |  |  |  |
| 1. I have trouble with constipation |  |  |  |  |
| 1. My heart beats faster than usual |  |  |  |  |
| 1. I get tired for no reason |  |  |  |  |
| 1. My mind is as clear as it used to be |  |  |  |  |
| 1. I find it easy to do the things I used to |  |  |  |  |
| 1. I am restless and can't keep still |  |  |  |  |
| 1. I feel hopeful about the future |  |  |  |  |
| 1. I am more irritable than usual |  |  |  |  |
| 1. I find it easy to make decisions |  |  |  |  |
| 1. I feel that I am useful and needed |  |  |  |  |
| 1. My life is pretty full |  |  |  |  |
| 1. I feel that others would be better off if I were dead |  |  |  |  |
| 1. I still enjoy the things I used to do |  |  |  |  |
